# Effects of an educational planetary plate graphic on meat consumption in a Stanford University dining hall: a randomized controlled trial

**DOI:** 10.1186/s40795-023-00764-3

**Published:** 2023-09-25

**Authors:** Alessandra L. Marcone, Gary L. Darmstadt, Ghislaine Amsler Challamel, Maya B. Mathur, Christopher D. Gardner

**Affiliations:** 1 Stanford Prevention Research Center, Stanford University School of Medicine, Stanford, CA USA; 2 Department of Pediatrics, Stanford University School of Medicine, Stanford, CA USA; 3 Stanford Residential and Dining Enterprises, Stanford University, Stanford, CA USA; 4 Quantitative Sciences Unit, Stanford University School of Medicine, Stanford, CA USA

**Keywords:** Planetary Health Plate, Educational signage, Behavior change, Environmental sustainability, Direct behavioral outcome

## Abstract

**Background:**

Assess the impact of an educational Planetary Health Plate (PHP) graphic on meat-related dietary choices of Stanford University dining hall patrons using a randomized controlled trial crossover design. All patrons entering the dining hall during study periods were enrolled as participants. Control, *n* = 631; PHP, *n* = 547.

**Methods:**

Compare dietary behavior without signage to behavior while exposed to PHP during four equivalent dinner meals. The primary outcome was total meat-dish weight adjusted for the number of people entering the dining hall. Secondary outcomes included the number of meat-dish servings and average meat-dish serving weight. Analysis using T-tests, Poisson generalized linear model.

**Results:**

Differences in total meat-dish weight, (1.54 kg; 95% Confidence Interval [CI] = -4.41,1.33; *P* = *.19*) and average meat-dish serving weight (0.03 kg; 95% CI = 0.00, 0.06; *P* = *.07*) between PHP and control patrons did not reach significance. The rate at which PHP patrons took meat was significantly lower (Incidence Rate Ratio 0.80; 95% CI = 0.71, 0.91; *P* < *.001*).

**Conclusion:**

Exposure to an educational plate graphic decreased the proportion of patrons taking meat but had no impact on total meat consumption or meat-dish serving weight. Statistical methods used in this study may inform future investigations on dietary change in the dining hall setting. Further research on the role of educational signage in influencing dietary behavior is warranted, with an aim to improve human health and environmental sustainability.

**Trial registration:**

ClinicalTrials.gov, NCT05565859, registered 4 October 2022

## Background

Current shifts in global eating trends toward a more westernized diet pose a danger to both human health and environmental sustainability, as increased meat consumption exacerbates the burden of non-communicable diseases, food insecurity, and environmental degradation [1–3]. The Lancet recently published the EAT Lancet Commission report that proposed a healthy and sustainable Planetary Health reference diet designed to help maintain planetary health, human health, and the health of systems upon which planetary and human health depend [4]. The diet was constructed upon multi-disciplinary expert assessment of currently available evidence on the nutritional and environmental impacts of varying food groups. However, there is relatively little research on strategies to shift consumer dietary behavior to better resemble this diet.

In the Commission report, particular emphasis was placed upon minimizing consumption of red meat and dairy products, as these food groups were identified as the most deleterious to both the environment and human health – increasing an individual’s risk for cardiovascular disease, stroke, diabetes and some cancers – with an estimated 14% increase of mortality per 0.5 servings (14 g) of meat consumed [4, 5]. The population of North America consumes more meat – 275 g/day – than any other region on the planet, with average daily consumption amounting to nearly double the recommendation of 113 g/day from the USDA [6].

The agricultural sector is one of the biggest contributors to anthropogenic climate change [1]. Agriculture contributes approximately 15% of global greenhouse gas emissions annually, with more a than a third of these emissions coming from meat and dairy cattle farming [4]. Anthropogenic climate change has the potential to dramatically harm human health and food systems, creating global food insecurity. Food availability, access, utilization, and stability, the pillars of food insecurity, are all vulnerable to extreme weather events and long-term, gradual climate risks [7]. Preserving the future of public health thus requires mitigating human impacts on the environment.

In many other sectors of human behavior, such as online purchasing of products or hotel selection, increased availability of information has led to changed consumer behavior [8]. While some research supports nutritional information as a catalyst for consumer behavior change, [9] including two studies conducted in a dining hall setting, [10, 11] other data fail to support that nutritional labeling leads to improved health decisions in consumers [12]. Each of these studies used subjective outcome measures to assess shifts in dietary behavior. Research assessing dietary change using direct behavior outcomes is rare yet necessary to avoid social desirability bias and identify effective interventions for dietary change [13].

Research suggests that images may be the most influential form of media in communicating the effects of climate change in comparison to textual information [14]. Images are also more accessible and easily distributed than other modes of communication such as videos and virtual reality which require more advanced technology and higher costs, thus limiting accessibility. Furthermore, pairing images with key informative text has been shown to increase content retention and reading comprehension in the classroom [15].

**Fig. 1:**
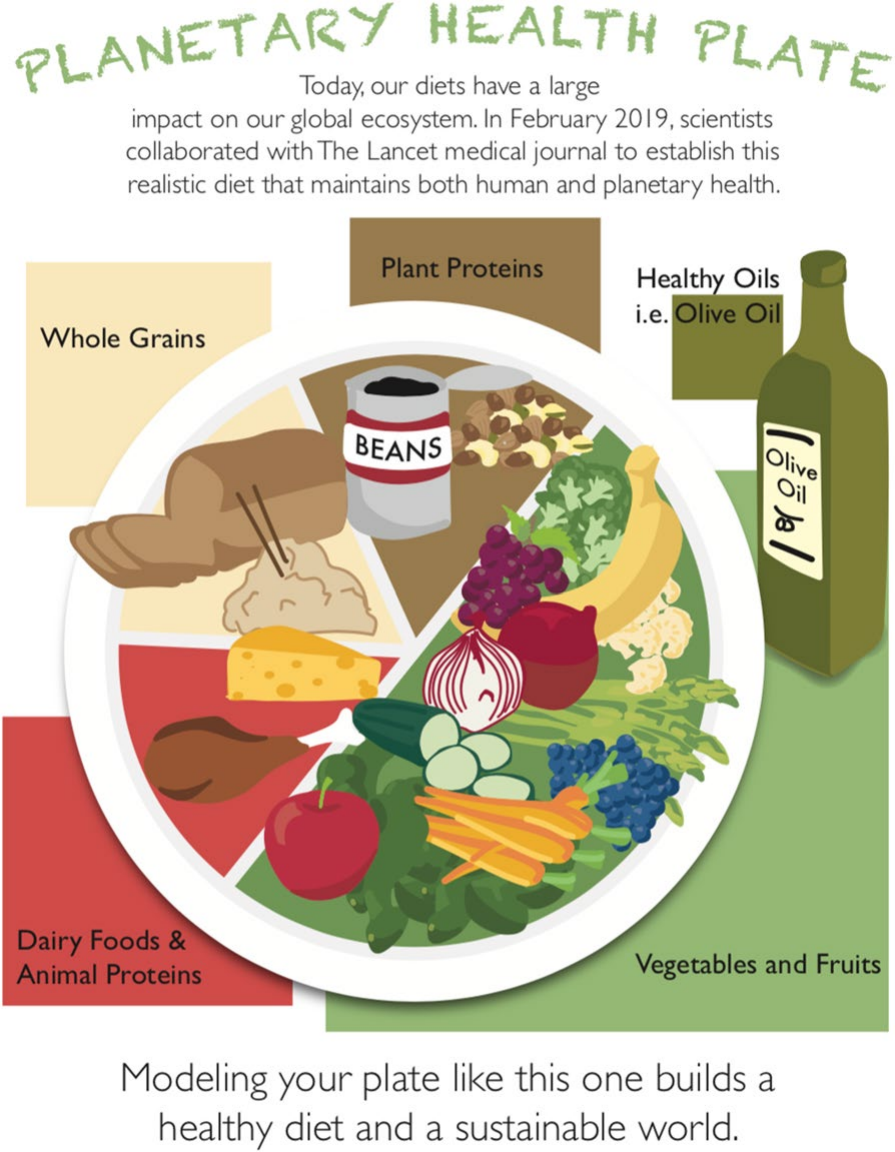
Planetary Health Plate (PHP) graphic featuring diet based on the EAT Lancet Planetary Health Diet

The objective of this research was to determine if adding a plate graphic depicting the components of the EAT Lancet Planetary Health diet (Fig. 1) to food labels in Stanford University dining halls would lead dining hall patrons to make dietary decisions that better resemble the Planetary Health diet in comparison to a no signage control group. Using the amount of meat taken by diners as a proxy for alignment with the Planetary Health diet, we hypothesized that presenting students with a plate graphic featuring the healthy reference diet would decrease objective measures of the amount of meat taken.

## Methods

### Study site, design, and participants

An established collaboration between researchers, chefs, and dining operators at Stanford University (Menus of Change University Research Collaborative) [16] provided an opportunity to nest this research into the everyday experience of dining hall patrons. All participants were patrons of Florence Moore Dining Hall on the Stanford campus where all data were collected. There were no selection, inclusion, or exclusion criteria; all students who entered the Dining Hall were enrolled and given an information sheet which explained the purpose and observational procedures of the study. Patrons were largely undergraduate students, with students from all four undergraduate class years well represented. During an average dinner meal at Florence Moore Dining Hall, approximately 150 patrons enter the dining hall. The Stanford undergraduate student population at the time of the study was reported to be 50% female and comprised of 32% White, 23% Asian, 17% Hispanic or Latino, 9% two or more races, 7% Black or African American, 1% American Indian or Alaskan native, < 1% Native Hawaiian or other Pacific Islander, and < 1% unknown. Ethnic data on international students, who comprise 11% of the undergraduate population, were not available [17].

This investigation was designed as a crossover randomized control trial with two phases: (1) a Planetary Health Plate (PHP) intervention phase where signage promoting the planetary health diet was posted and (2) a control phase with signage as usual, including dish labels listing ingredients and allergens. Stanford Dining serves food on a four-week menu cycle; this pattern results in several academic weeks that are intended to be identical in food served. To standardize the conditions and limit potential confounding, the experimental and control phases were each randomized to one of the weeks throughout the quarter when the same, “week two” menu was to be served. The phases were randomized by drawing equally sized pieces of paper from a hat. Following randomization, data were collected for the control phase during week two of the academic winter quarter and data with the PHP posted were collected during week six of the academic winter quarter. There was a last-minute change in the Tuesday menu for the scheduled sixth week of the PHP phase that could have made menu items non-comparable between the control and intervention periods, so a post hoc protocol adjustment was made to restore the comparability of menu items. The dining management staff agreed to change the Tuesday menu of week seven to the originally planned menu that should have been served during the Tuesday in week six. The plate sign was left posted throughout the weekend and was taken down following Tuesday’s data collection.

### Graphic design and development

A PHP graphic was developed based on the healthy reference diet proposed by the EAT Lancet Commission. The graphic was designed to capture the food groups and proportions of food groups promoted by the Planetary Health diet. For the purpose of accessibility and readability, categories on the graphic were simplified. The category, “added sugars,” was left out due to the small proportion size and its potential to confuse dining hall patrons. “Unsaturated plant oils” was changed to “healthy oils” to increase accessibility of phrasing to a less scientific audience. Similarly, “animal-source protein” was simplified to “animal proteins” and this label included “dairy foods” in order to increase readability of the sign from a typical distance.

The sign also included educational text to contextualize the information on top of the sign: “Today, our diets have a large impact on our global ecosystem. In February 2019, scientists collaborated with The Lancet medical journal to establish this realistic diet that maintains both human and planetary health.” And on the bottom of the sign “Modeling your plate like this one builds a healthy diet and a sustainable world”. The graphic was approved for use by Stanford Residential and Dining Services.

### Data collection

**Fig. 2:**
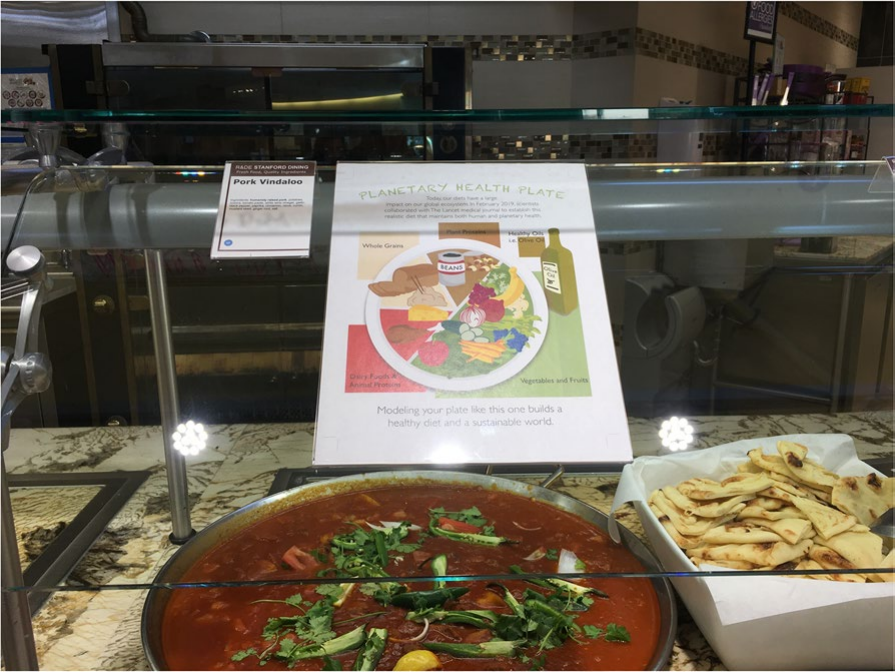
Placement of signs on sneeze guard in front of animal protein options

During weeks two and six of the 2020 winter 10-week academic quarter, data were collected during dinner meals Monday through Thursday night from 6:00 pm to 7:15 pm. The PHP was posted throughout the entire assigned week from Sunday night until Thursday evening. Placing the sign on Sunday night ensured that the signs were present for all meals on Monday. This allowed researchers to maintain consistency with all other days during the collection week when the sign was continually posted. The sign was placed on top of the sneeze guard directly in patrons’ line of sight to meat protein options (Fig. 2).

Data were collected during study meals by research assistants who received a 15-min training session and were supervised by the research director [AM], who was present during all data collection periods. On each data collection day, two different types of meat dishes—including a meat, sauce, and garnish—were provided by the Dining Hall and there were two stations with each type. In addition to meat dishes, the dining hall also offered plant protein options like “miso tofu” and “hongos guisados stew.” Research assistants weighed each serving tray of meat as it came from the kitchen and again before returning the used serving tray to the kitchen, to determine the total amount of meat taken from the tray. All weight measurements were taken in pounds (lbs) to the nearest tenth using a LEM Products 1167 Stainless Steel Digital Scale (330-Pound Capacity), the standard scale metric used by Stanford Dining to track food, and then converted to kilograms (kg). Tally counters were used to count the number of patrons who took meat from the tray. If a patron took from both provided dishes (i.e., both types of meat), they were counted as a patron for each dish. If a patron took from both stations of the same dish, they were counted as two patrons for the dish. Qualitative notes about patron behavior and possible deviations from data collection protocol were recorded on data collection sheets. After each dinner collection period, Stanford Dining provided the total number of people who entered the dining hall during the designated dinner periods on each of the data collection days. This was based on the number of unique identification card swipes into the dining hall during dinner times.

**Fig. 3:**
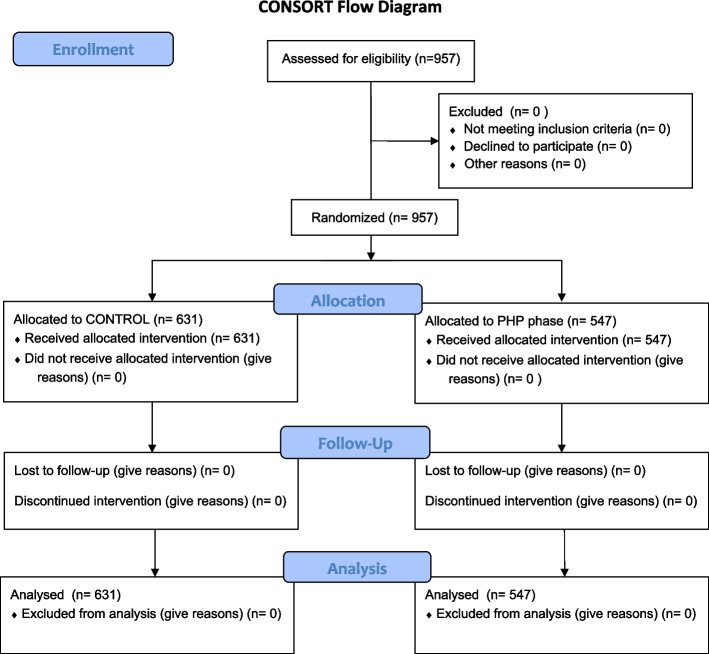
Visualization of allocation of participants into interventions by the CONSORT flow chart for Randomized Controlled Trials

Completed versions of the CONSORT checklist and flow chart for Randomized Controlled Trials were both completed following study completion and are included for reference (Fig. 3).

### Data analysis

The data collection process involved hierarchically sampling all menu items containing meat offered during the dinner meal of a given day (e.g., all of Monday’s dinner meat menu items), then sampling servings taken from both physical stations for each of the two meat dishes offered on each day. We expected servings taken from different meat types on the same day to be statistically dependent (e.g., because patrons might choose to take only one of the two options) and also for stations to be statistically dependent (e.g., because patrons taking a dish from one station would be less likely to also take a dish from the other station). Because we did not have individual subject-level data, we minimized such problems of dependence by aggregating data to the phase-day level, such that the unit of analysis was total meat weight or servings taken on each day of each phase. Thus, we analyzed eight observations [four day-menus (Monday-Thursday) x two phases (control, PHP)].

We considered three outcome measures: (1) total meat-dish weight (the total weight in kilograms of meat dishes that patrons took on a given day adjusted for the number of people who entered the dining hall); (2) the number of meat-dish servings (the number of tallied servings taken from meat dishes on a given day adjusted for the number of people who entered the dining hall); and (3) average meat-dish serving weight (calculated for a given day as the total weight divided by the number of meat-dish servings). Total meat-dish weight was the primary outcome with outcomes (2) and (3) being secondary.

For the two continuous outcomes (total meat weight and average meat serving weight), we calculated pairwise differences (intervention minus control) for each of the four days. These pairwise differences represent average intervention effects while holding constant any differences in day-menu offerings. We then conducted a t-test on these pairwise differences. For the count outcome (number of meat-dish servings), we used a Poisson generalized linear model to regress servings on intervention, a fixed effect of day-menu, and an offset term of log-swipes. The offset term serves as a denominator for the number of servings, thus accounting for the fact that more servings would be expected on days with more people. The Poisson model estimated the intervention effect as an incidence rate ratio (IRR), which represents the multiplicative change in the rate of patrons’ taking meat-dish servings on intervention versus control days, again holding constant differences in day-menu offerings. An IRR less than 1 would indicate a reduction in meat-dish servings on intervention versus control days. The t-test on paired day-menu differences is equivalent to linear regression with fixed effects of treatment and day. Because total meat-dish weight is a sum of many random variables (i.e., the meat-dish weight taken by each person on a given day), the Central Limit Theorem suggests that meat-dish weight would be normally distributed. With normally distributed outcomes, t-tests are statistically valid even with few (four pairs) observations.

### Ethical approval and trial registration

Expedited ethical approval for the study was obtained from Stanford Institutional Review Board-6 (protocol #52,729), which waived the need for informed consent. The trial was registered at Clinicaltrials.gov protocol NCT05565859 on 04/10/2022.

## Results

Over both phases (PHP and control weeks), 957 patrons were recorded to have taken meat. There were 631 and 547 people who entered the dining hall over the four meals for the control phase and the four meals for the PHP phase, respectively (Fig. 3). There were 560 patrons who took from a meat dish during the control phase and 397 patrons who took meat during the PHP phase. Qualitative notes taken by researcher assistants throughout data collection noted that most patrons did not appear to pay attention to the posted signage despite its position directly in line-of-sight of meat dishes.

Research assistants also noted unintended inconsistencies in the cuts of meats. Some cuts of chicken and pork served during the PHP phase were notably larger than the cuts served during equivalent days during the control phase. This discrepancy was discussed with the chefs; however, entrees had already been prepared at the time of discussion and could not be adjusted. No weight comparisons of the cuts of meats on different days were available, but post hoc sensitivity analysis was conducted to explore the potential influence of days for which observations suggested that larger cuts of meat were served.

For a given menu on a given day, the total meat-dish weight of PHP patrons was 1.54 kg lower [95% Confidence Interval (CI) -4.41 kg to 1.33 kg] than for control patrons; an absolute difference that did not reach statistical significance (*P* = *0.19)*. However, the rate at which patrons took meat-dish servings, based on Poisson regression analysis, was significantly reduced by 20% [IRR 0.80 (95% CI 0.71–0.91) *P* < *0.001*] in the PHP vs. the control phase. The explanation for total meat not reaching statistical significance but servings being lower for PHP was that the average meat-dish serving weight was modestly higher by 0.03 kg (95% CI -0.004 kg to 0.06 kg) in PHP compared to control patrons, a difference that did not reach statistical significance (*P* = *0.07*).

In the sensitivity analyses that excluded the two Thursdays on which the meat cut sizes may have differed systematically between phases, the total meat-dish weight was significantly less for PHP vs. control patrons [-2.63 kgs, (95% CI -4.06 to -1.20), *P* = *0.03*]. The IRR for meat-dish servings in this analysis was slightly closer to the null than in the main analyses [IRR 0.87 (95% CI 0.72 to 1.05), *P* = *0.14*], and the difference in average serving weight was smaller – a non-significant difference (*P* = *0.40*) of 0.01 kg (95% CI -0.11 to 0.14) higher for PHP than control patrons (Table 1).

**Table 1:** Summary of results comparing PHP to control by total meat-dish weight, rate at which patrons selected meat, and average meat-dish serving weight

**Outcome measure**	**Difference between PHP and control**	**Statistical significance by CI or IRR**
Difference in total meat-dish weight between PHP patrons and control patrons	-1.54 kg	95% CI (-4.41 kg to 1.33 kg), *P* = 0.19
Difference in the rate at which patrons took meat-dish servings between PHP and control phases	-20%	IRR 0.80 (95% CI 0.71–0.91), *P* < 0.001
Difference in average meat-dish serving weight between PHP patrons and control patrons	+ 0.03 kg	95% CI (-0.004 kg to 0.06 kg), *P* = 0.07
Difference in total meat-dish weight between PHP patrons and control patrons—Excluding days potentially displaying different meat cut size	-2.63 kg	95% CI (-4.06 kg to -1.2 kg), *P* = 0.03
Difference in the rate at which patrons took meat-dish servings between PHP and control phases—Excluding days potentially displaying different meat cut size	-13%	IRR 0.87 (95% CI 0.72 to 1.05), *P* = 0.14
Difference in average meat-dish serving weight between PHP patrons and control patrons—Excluding days potentially displaying different meat cut size	+ 0.01 kg	95% CI (0.11 kg to 0.14 kg). *P* = 0.40

For continuous outcomes (total meat weight and average meat serving weight), pairwise differences were calculated and a t-test conducted on those differences. For the count outcome (number of meat-dish servings), a Poisson generalized linear model was used to estimate the intervention effect as an IRR and upon which t-tests were then conducted

*CI* Confidence Interval, *IRR* incidence rate ratio, *PHP* planetary health plate

## Discussion

In this study, the main hypothesis was not supported, as the primary outcome of total meat-dish weight was not significantly different in the main analysis comparing PHP and control phases. We found, however, that patrons took meat dishes at a 20% lower rate while the PHP was posted, while average serving weight was similar. It is possible that individuals who typically take less meat were dissuaded from taking any by exposure to the PHP. Furthermore, due to observations of a potential systematically higher meat cut size during some PHP test periods, we conducted a sensitivity analysis which excluded those data. Sensitivity analysis revealed a significantly lower total meat-dish weight by 2.63 kg for PHP patrons, thus supporting our main hypothesis; the rate of meat dish servings and the average serving weight were similar for PHP and control patrons in the sensitivity analysis.

Overall, it appears that this intervention at the point of food selection in university dining halls may have been insufficient or too late to make substantial impacts on patrons’ choices. Their focus in the moment on the food decision process may have distracted them from learning from posted signs, as suggested by the apparent lack of attentiveness to the PHP observed during meal selection. Peterson et al. found positive changes in patron perception of healthy foods after posting signage at the point of selection in a university dining hall [10]. Unlike our investigation, the Peterson study did not post signs exclusively at the point of selection. Large signs, table tents, flyers and colorful photographs were all used. A limitation of the Peterson et al. study, however, is that they assessed dietary change using a self-reported survey method, with a 38% response rate from dining patrons [10]. In contrast, our study utilized objective measures of dietary change, measuring the total weight of serving dishes for meat and tallying patrons. While there is a body of literature demonstrating that “point of purchase” signage can be effective in changing consumer choices [18, 19], there are important differences between point of purchase in a grocery store and point of selection in a university dining hall where direct exchange of money does not play a role and patrons are taking buffet food that they plan to eat immediately afterward. Furthermore, our qualitative observation that patrons did appear to pay significant attention to signs during data collection indicates further research may be helpful to determine how patrons integrate point of selection information into meal decisions. However, the nature of the message might also play a key role in influencing patrons’ food choices. Turnwald et al. at the same university showed that taste-centric messaging posted at the point of selection increased the selection and consumption of plant-based dishes, whereas health-centric messages did not have any effect [20].

There are several possible confounding variables that may have contributed to the results observed. We investigated one potential confounder – inconsistency in meat cut sizes – using a sensitivity analysis eliminating days with suspected meat cut issues. Although dining hall chefs helped ensure that dishes were consistent in this manner between the two weeks, the size of pieces provided during each phase was not necessarily the same. The sensitivity analysis suggested that total meat taken by PHP patrons was significantly less than control patrons, but the magnitude of the meat-dish servings was diminished slightly and did not reach statistical significance, and thus results remained mixed.

Another variable that we could not control for was time. The second academic week differs from the sixth academic week in both midterm frequency and volume of class work. Academic literature points towards a complex relationship between stress and individual eating habits, with evidence that stress can either increase or decrease dietary intake [21, 22]. As stress appears to be related to variability in eating behavior, it is possible that the stress of week six midterms and assignments, during the PHP assessment, had a divergent effect on patron eating behavior, leading some patrons to eat more and others to eat less. This could potentially contribute to the increase in serving size and decrease in proportion of patrons taking meat.

An additional limitation of our study was the inability to collect individual level data, which hindered the power of our analysis. For future trials, a sample size larger than the eight data points collected is recommended. Due to the lack of availability of appropriate variance data for the number or percent of individuals that take from meat dishes in a dining hall setting, we were unable to conduct a sample size calculation during development of our study. The data collected in this study will now make it possible to design a future trial, including appropriate sample size, more precisely. This investigation would have been further strengthened by expanding to assessment of all foods in the dining hall rather than focusing only on meat. Such an experiment would allow for calculation of the percentage of food taken from each category of the PHP and more effectively capture shifts in food selection.

An important strength of this study was the use of direct behavioral outcomes to assess changes in dietary behavior rather than self-report measures which can be subject to social desirability bias, or an artificial online environment. Objective outcome measures are crucial for assessing effective interventions for food selection and have rarely been used in practice [15]. Nonsignificant results collected in a living laboratory provide valuable context to studies conducted using MTurk or other digital tools that do not reflect the complexity of real life [23].

## Conclusion

While we found a nonsignificant decrease in total meat-dish weight, our sensitivity analysis gives interesting clues and learnings for further research in dining halls used as living laboratories. Communications and marketing strategies to promote healthier and more sustainable diets need to be further evaluated in real-life settings as they are low-cost, low-labor, and easily implemented in dining operations. More research is warranted on use of multi-pronged approaches in further attempts to motivate sustainable dietary change.

This study also establishes a statistical method for managing the inevitable dependence between the dishes and stations within a dining hall setting when meat consumption is measured at the aggregate rather than individual level. This approach may serve as a valuable resource for researchers working in a dining hall setting to assess the impact of nutrition education on dietary choices or gather baseline data eating behaviors.

Shifting dietary behaviors meaningfully has been and will continue to be challenging. Evidence increasingly suggests that substantive and sustained dietary behavior changes require combinations of multiple approaches [24]. The preliminary findings of this point-of-selection signage intervention suggest several potential areas of follow-up. A follow-up study could identify students who prioritize sustainability and examine why these students initially began factoring issues of sustainability into their dietary decisions. With more comprehensive information about why students shift their priorities, more effective strategies could potentially be developed to guide student behavior change. Future interventions might be made more potent by appealing to additional reasons for reducing meat consumption, such as concerns about animal welfare and health [15, 25]. Another potential future direction is investigation of how presentation of different sized cuts of meats affects meat consumption. Perhaps there is a particular serving size that is large enough that patrons only take one piece, but small enough to reduce general meat consumption.

Despite challenges in shifting human behavior, it remains imperative to advance our understanding of how to promote diets that enhance health, food security and environmental sustainability, such as *The Lancet* Planetary Health diet. The health of the planet and humankind ultimately depends on it.

## Data Availability

The dataset supporting the conclusions of this article is available in the OSF repository, https://osf.io/7s8wq/?view_only=e3c057b7bc1f48c8841d0a1b01ffdb0e.

## Abbreviations

PHP: Planetary Health Plate
IRR: Incidence rate ratio
CI: Confidence interval
lb: Pound
kg: Kilograms
